# Aqua­cyanido{6,6′-dimeth­oxy-2,2′-[1,2-phenyl­enebis(nitrilo­methanylyl­idene)]diphenolato}cobalt(III) acetonitrile hemisolvate

**DOI:** 10.1107/S160053681105330X

**Published:** 2011-12-17

**Authors:** Yang Lin, Guang-Feng Hou, Guang-Ming Li, Peng-Fei Yan

**Affiliations:** a School of Chemistry and Materials Science, Heilongjiang University, Harbin 150080, People’s Republic of China

## Abstract

In the title complex, [Co(C_22_H_18_N_2_O_4_)(CN)(H_2_O)]·0.5CH_3_CN, the Co^III^ cation is *N*,*N*′,*O*,*O*′-chelated by a 6,6′-dimeth­oxy-2,2′-[1,2-phenyl­enebis(nitrilo­methanylyl­idene)]diphenolate dianion, and is further coordinated by a cyanide anion and a water mol­ecule in the axial sites, completing a distorted octa­hedral coordination geometry. In the crystal, pairs of bifurcated O—H⋯(O,O) hydrogen bonds link adjacent mol­ecules, forming centrosymmetric dimers. The acetonitrile solvent mol­ecule shows 0.5 occupancy.

## Related literature

For the synthesis, see: Costes *et al.* (2000 ▶). For related complexes with a similar ligand, see: Lin *et al.* (2011 ▶). For bond-valence calculations, see: Spek (2009 ▶).
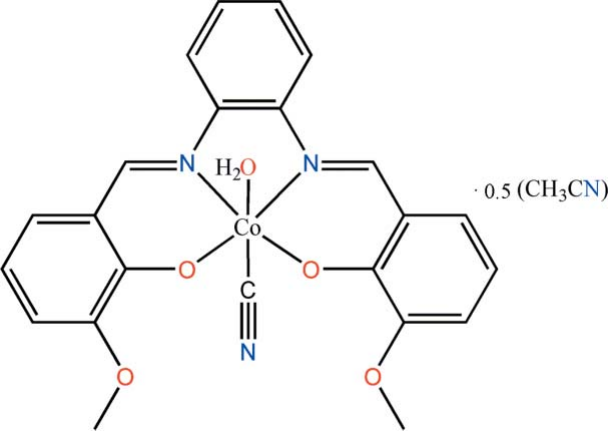


## Experimental

### 

#### Crystal data

[Co(C_22_H_18_N_2_O_4_)(CN)(H_2_O)]·0.5C_2_H_3_N
*M* *_r_* = 497.95
Triclinic, 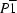

*a* = 8.6487 (17) Å
*b* = 11.689 (2) Å
*c* = 12.229 (2) Å
α = 112.10 (3)°
β = 102.30 (3)°
γ = 97.85 (3)°
*V* = 1086.6 (5) Å^3^
*Z* = 2
Mo *K*α radiation
μ = 0.83 mm^−1^
*T* = 293 K
0.23 × 0.21 × 0.16 mm

#### Data collection

Rigaku R-AXIS RAPID diffractometer
Absorption correction: multi-scan (*ABSCOR*; Higashi, 1995 ▶) *T*_min_ = 0.831, *T*_max_ = 0.878
10646 measured reflections
4911 independent reflections
3570 reflections with *I* > 2σ(*I*)
*R*_int_ = 0.030

#### Refinement

*R*[*F* ^2^ > 2σ(*F* ^2^)] = 0.049
*wR*(*F* ^2^) = 0.158
*S* = 1.05
4911 reflections
319 parameters
16 restraints
H-atom parameters constrained
Δρ_max_ = 0.52 e Å^−3^
Δρ_min_ = −0.89 e Å^−3^

### 

Data collection: *RAPID-AUTO* (Rigaku, 1998 ▶); cell refinement: *RAPID-AUTO*; data reduction: *CrystalClear* (Rigaku/MSC, 2002 ▶); program(s) used to solve structure: *SHELXTL* (Sheldrick, 2008 ▶); program(s) used to refine structure: *SHELXTL*; molecular graphics: *SHELXTL*; software used to prepare material for publication: *SHELXTL*.

## Supplementary Material

Crystal structure: contains datablock(s) I, global. DOI: 10.1107/S160053681105330X/xu5401sup1.cif

Structure factors: contains datablock(s) I. DOI: 10.1107/S160053681105330X/xu5401Isup2.hkl

Additional supplementary materials:  crystallographic information; 3D view; checkCIF report

## Figures and Tables

**Table 1:** Selected bond lengths (Å)

Co1—O1	1.884 (2)
Co1—O3	1.884 (2)
Co1—O5	2.030 (2)
Co1—N1	1.890 (2)
Co1—N2	1.885 (3)
Co1—C23	1.858 (3)

**Table 2:** Hydrogen-bond geometry (Å, °)

*D*—H⋯*A*	*D*—H	H⋯*A*	*D*⋯*A*	*D*—H⋯*A*
O5—H51⋯O1^i^	0.85	2.18	2.913 (3)	145
O5—H51⋯O2^i^	0.85	2.24	2.959 (3)	142
O5—H52⋯O3^i^	0.85	2.28	2.926 (3)	133
O5—H52⋯O4^i^	0.85	2.10	2.883 (3)	153

Symmetry code: (i) 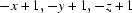.

**Fig. 1.**
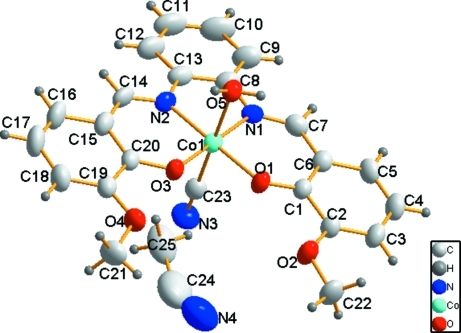
The molecular structure of the title compound, showing 50% probability displacement ellipsoids.

## supplementary crystallographic information

### Comment

Transition metal complexes with spectroscopic and magnetic properties are currently of considerable interest. In continuation of the studies of salen type transition metal complexes, we present here the synthesis and the crystal structure of the title compound. The similar structure has been reported by us, both of which are unexpected products (Lin *et al.*, 2011).

The bond-valence calculation (Spek, 2009) indicate that the cobalt is in +3 states, which should be produced by LiTCNQ oxidating Co(II) atom [TCNQ = 2,2'-(2,5-cyclohexadiene-1,4-diylidene)bis-propanedinitrile], meanwhile, the TCNQ decompose to produce cyandio group. The Co(III) ion is six-coordinated by two imino nitrogen atoms and one nitrogen atom from the decomposition of LiTCNQ and two phenolate oxygen atoms from the ligand and one oxygen from the hydrate group (Fig. 1). The Co—N bond distances range from 1.886 (3) Å to 1.890 (2) Å and the Co—O bond distances range from 1.884 (2) Å to 2.031 (2) Å, in accordance with the reported values. The axially coordinated hydrate oxygen atoms form H-bonding with four oxygen atoms of the adjacent ligand constructing dimer (Table 1). One acetonitrile molecule is co-crystallized with the dimer.

### Experimental

A solution of CoL (0.008 g, 0.01 mmol) (L = N,N'-bis(2-oxy-3-methoxybenzylidene)-1,2-diaminobenzene) (Costes *et al.*, 2000) in 30 ml of CH_3_CN was added dropwise to a solution of LiTCNQ (0.006 g, 0.03 mmol) [TCNQ = 2,2'-(2,5-cyclohexadiene-1,4-diylidene)bis-propanedinitrile] in 20 mL of H_2_O solution. The reaction was carried out under oxygen-free nitrogen, using standard Schlenk techniques and degassed solvents. Red-brown single crystals suitable for X-ray determination were obtained in seven days. Elemental Anal. Calc. for C_48_H_43_N_7_O_10_Co_2_: C, 58.72; H, 4.41; N, 8.56 wt%, Found: C, 58.66; H, 4.50; N, 8.59 wt%.

### Refinement

H atoms were placed in calculated positions with C—H = 0.93–0.96 Å and O—H = 0.85 Å, and refined in riding mode with U_iso_(H) = 1.5U_eq_(C,O) for methyl and water H atoms, and 1.2U_eq_(C) for aromatic H atoms. The disordered acetonitrile solvatete molecule is treated with 50% occupancy, and the bonds of which is restricted by a series of commonds: dfix 1.15 0.001 n4 c24, dfix 1.47 0.001 c24 c25, and dfix 2.62 0.001 n4 c25 to keep the linear configuration.

### Crystal data

**Table:**

[Co(C_22_H_18_N_2_O_4_)(CN)(H_2_O)]·0.5C_2_H_3_N	*Z* = 2
*M**_r_* = 497.95	*F*(000) = 514
Triclinic, *P*1	*D*_x_ = 1.522 Mg m^−^^3^
Hall symbol: -P 1	Mo *K*α radiation, λ = 0.71073 Å
*a* = 8.6487 (17) Å	Cell parameters from 8343 reflections
*b* = 11.689 (2) Å	θ = 3.2–27.5°
*c* = 12.229 (2) Å	µ = 0.83 mm^−^^1^
α = 112.10 (3)°	*T* = 293 K
β = 102.30 (3)°	Block, red-brown
γ = 97.85 (3)°	0.23 × 0.21 × 0.16 mm
*V* = 1086.6 (5) Å^3^	

### Data collection

**Table:**

Rigaku R-AXIS RAPID diffractometer	4911 independent reflections
Radiation source: fine-focus sealed tube	3570 reflections with *I* > 2σ(*I*)
graphite	*R*_int_ = 0.030
ω scan	θ_max_ = 27.5°, θ_min_ = 3.2°
Absorption correction: multi-scan (*ABSCOR*; Higashi, 1995)	*h* = −11→11
*T*_min_ = 0.831, *T*_max_ = 0.878	*k* = −15→15
10646 measured reflections	*l* = −15→15

### Refinement

**Table:**

Refinement on *F*^2^	Primary atom site location: structure-invariant direct methods
Least-squares matrix: full	Secondary atom site location: difference Fourier map
*R*[*F*^2^ > 2σ(*F*^2^)] = 0.049	Hydrogen site location: inferred from neighbouring sites
*wR*(*F*^2^) = 0.158	H-atom parameters constrained
*S* = 1.05	*w* = 1/[σ^2^(*F*_o_^2^) + (0.1004*P*)^2^] where *P* = (*F*_o_^2^ + 2*F*_c_^2^)/3
4911 reflections	(Δ/σ)_max_ < 0.001
319 parameters	Δρ_max_ = 0.52 e Å^−^^3^
16 restraints	Δρ_min_ = −0.89 e Å^−^^3^

### Special details

**Table:**

Geometry. All esds (except the esd in the dihedral angle between two l.s. planes) are estimated using the full covariance matrix. The cell esds are taken into account individually in the estimation of esds in distances, angles and torsion angles; correlations between esds in cell parameters are only used when they are defined by crystal symmetry. An approximate (isotropic) treatment of cell esds is used for estimating esds involving l.s. planes.
Refinement. dfix 1.15 0.001 n4 c24 dfix 1.47 0.001 c24 c25 dfix 2.62 0.001 n4 c25 isor 0.01 c24 n4 dfix 1.50 0.01 c24 c25 Refinement of F^2^ against ALL reflections. The weighted R-factor wR and goodness of fit S are based on F^2^, conventional R-factors R are based on F, with F set to zero for negative F^2^. The threshold expression of F^2^ > 2sigma(F^2^) is used only for calculating R-factors(gt) etc. and is not relevant to the choice of reflections for refinement. R-factors based on F^2^ are statistically about twice as large as those based on F, and R- factors based on ALL data will be even larger.

### Fractional atomic coordinates and isotropic or equivalent isotropic displacement parameters (Å^2^)

**Table:**

	*x*	*y*	*z*	*U*_iso_*/*U*_eq_	Occ. (<1)
C1	0.5411 (4)	0.7915 (3)	0.5742 (3)	0.0327 (6)	
C2	0.6334 (4)	0.8623 (3)	0.6996 (3)	0.0366 (7)	
C3	0.6087 (4)	0.9779 (3)	0.7700 (3)	0.0438 (8)	
H3	0.6712	1.0227	0.8518	0.053*	
C4	0.4896 (4)	1.0283 (3)	0.7190 (3)	0.0474 (8)	
H4	0.4720	1.1057	0.7673	0.057*	
C5	0.4008 (4)	0.9649 (3)	0.6001 (3)	0.0421 (7)	
H5	0.3222	0.9992	0.5671	0.051*	
C6	0.4253 (4)	0.8455 (3)	0.5234 (3)	0.0364 (6)	
C7	0.3364 (4)	0.7893 (3)	0.3972 (3)	0.0400 (7)	
H7	0.2612	0.8304	0.3715	0.048*	
C8	0.2706 (4)	0.6445 (3)	0.1871 (3)	0.0416 (7)	
C9	0.1671 (4)	0.7046 (4)	0.1359 (4)	0.0521 (9)	
H9	0.1397	0.7759	0.1870	0.063*	
C10	0.1057 (5)	0.6584 (5)	0.0103 (4)	0.0650 (12)	
H10	0.0359	0.6981	−0.0235	0.078*	
C11	0.1465 (5)	0.5535 (5)	−0.0663 (4)	0.0615 (11)	
H11	0.1054	0.5240	−0.1513	0.074*	
C12	0.2471 (4)	0.4922 (4)	−0.0186 (3)	0.0537 (9)	
H12	0.2745	0.4217	−0.0708	0.064*	
C13	0.3082 (4)	0.5363 (3)	0.1093 (3)	0.0419 (7)	
C14	0.4470 (4)	0.3753 (3)	0.1133 (3)	0.0427 (7)	
H14	0.3935	0.3316	0.0295	0.051*	
C15	0.5590 (4)	0.3186 (3)	0.1660 (3)	0.0423 (7)	
C16	0.5876 (5)	0.2028 (3)	0.0879 (3)	0.0520 (9)	
H16	0.5282	0.1638	0.0051	0.062*	
C17	0.6983 (5)	0.1489 (4)	0.1313 (4)	0.0605 (10)	
H17	0.7137	0.0726	0.0786	0.073*	
C18	0.7910 (5)	0.2064 (3)	0.2554 (4)	0.0504 (9)	
H18	0.8702	0.1698	0.2839	0.061*	
C19	0.7655 (4)	0.3164 (3)	0.3349 (3)	0.0392 (7)	
C20	0.6478 (4)	0.3764 (3)	0.2929 (3)	0.0358 (7)	
C21	0.9775 (4)	0.3347 (4)	0.5076 (4)	0.0573 (10)	
H21A	0.9401	0.2479	0.4942	0.086*	
H21B	1.0214	0.3869	0.5944	0.086*	
H21C	1.0605	0.3403	0.4675	0.086*	
C22	0.8491 (5)	0.8702 (4)	0.8620 (3)	0.0602 (10)	
H22A	0.9104	0.9501	0.8719	0.090*	
H22B	0.9226	0.8202	0.8785	0.090*	
H22C	0.7845	0.8849	0.9186	0.090*	
C23	0.6571 (4)	0.6832 (3)	0.3194 (3)	0.0396 (7)	
C25	0.8598 (12)	0.8846 (9)	0.1678 (9)	0.082 (3)	0.50
H25A	0.7929	0.8049	0.1538	0.123*	0.50
H25B	0.9149	0.8713	0.1053	0.123*	0.50
H25C	0.7926	0.9429	0.1647	0.123*	0.50
C24	0.9809 (11)	0.9376 (14)	0.2899 (9)	0.140 (6)	0.50
N4	1.0569 (11)	0.9860 (13)	0.3921 (9)	0.132 (4)	0.50
Co1	0.49216 (5)	0.58348 (4)	0.34297 (4)	0.03236 (16)	
N1	0.3503 (3)	0.6849 (2)	0.3133 (2)	0.0349 (5)	
N2	0.4128 (3)	0.4825 (3)	0.1717 (2)	0.0366 (6)	
N3	0.7556 (4)	0.7451 (3)	0.3036 (3)	0.0600 (9)	
O1	0.5684 (3)	0.68069 (19)	0.51481 (19)	0.0362 (5)	
O2	0.7461 (3)	0.8045 (2)	0.7402 (2)	0.0476 (6)	
O3	0.6284 (3)	0.4801 (2)	0.37473 (19)	0.0367 (5)	
O4	0.8432 (3)	0.3779 (2)	0.4577 (2)	0.0461 (6)	
O5	0.3077 (2)	0.4757 (2)	0.36607 (19)	0.0371 (5)	
H51	0.3288	0.4073	0.3676	0.056*	
H52	0.2908	0.5179	0.4343	0.056*	

### Atomic displacement parameters (Å^2^)

**Table:**

	*U*^11^	*U*^22^	*U*^33^	*U*^12^	*U*^13^	*U*^23^
C1	0.0382 (15)	0.0286 (14)	0.0302 (15)	0.0068 (12)	0.0109 (12)	0.0111 (12)
C2	0.0422 (16)	0.0335 (15)	0.0345 (16)	0.0119 (13)	0.0108 (13)	0.0138 (13)
C3	0.058 (2)	0.0314 (16)	0.0363 (18)	0.0084 (15)	0.0156 (15)	0.0079 (14)
C4	0.065 (2)	0.0344 (17)	0.048 (2)	0.0193 (16)	0.0244 (18)	0.0155 (15)
C5	0.0518 (19)	0.0372 (17)	0.049 (2)	0.0205 (15)	0.0229 (16)	0.0230 (16)
C6	0.0430 (16)	0.0322 (15)	0.0361 (17)	0.0079 (13)	0.0135 (13)	0.0159 (13)
C7	0.0390 (16)	0.0454 (18)	0.0448 (19)	0.0130 (14)	0.0130 (14)	0.0270 (16)
C8	0.0410 (16)	0.0450 (18)	0.0380 (18)	0.0022 (14)	0.0037 (14)	0.0235 (15)
C9	0.050 (2)	0.053 (2)	0.054 (2)	0.0070 (17)	0.0022 (17)	0.0319 (19)
C10	0.060 (2)	0.080 (3)	0.061 (3)	0.003 (2)	−0.003 (2)	0.052 (3)
C11	0.056 (2)	0.085 (3)	0.039 (2)	−0.005 (2)	−0.0026 (17)	0.038 (2)
C12	0.055 (2)	0.068 (2)	0.0322 (18)	−0.0007 (19)	0.0066 (15)	0.0227 (18)
C13	0.0401 (16)	0.0505 (19)	0.0326 (17)	−0.0005 (14)	0.0066 (13)	0.0208 (15)
C14	0.0464 (17)	0.0461 (18)	0.0222 (15)	−0.0018 (15)	0.0074 (13)	0.0058 (13)
C15	0.0538 (19)	0.0385 (17)	0.0315 (16)	0.0066 (15)	0.0183 (14)	0.0096 (14)
C16	0.075 (2)	0.0423 (18)	0.0342 (18)	0.0136 (18)	0.0233 (17)	0.0074 (15)
C17	0.088 (3)	0.048 (2)	0.050 (2)	0.027 (2)	0.038 (2)	0.0122 (18)
C18	0.061 (2)	0.0440 (19)	0.053 (2)	0.0235 (17)	0.0267 (18)	0.0189 (17)
C19	0.0439 (17)	0.0349 (16)	0.0412 (18)	0.0097 (14)	0.0190 (14)	0.0149 (14)
C20	0.0418 (16)	0.0329 (15)	0.0320 (16)	0.0058 (13)	0.0171 (13)	0.0104 (13)
C21	0.049 (2)	0.057 (2)	0.071 (3)	0.0225 (18)	0.0124 (19)	0.031 (2)
C22	0.065 (2)	0.063 (2)	0.0345 (19)	0.016 (2)	−0.0011 (17)	0.0092 (18)
C23	0.0424 (17)	0.0412 (17)	0.0363 (17)	0.0123 (14)	0.0093 (14)	0.0177 (14)
C25	0.113 (8)	0.082 (7)	0.094 (8)	0.041 (6)	0.059 (7)	0.059 (6)
C24	0.129 (9)	0.133 (9)	0.168 (10)	0.012 (7)	0.086 (8)	0.058 (7)
N4	0.077 (5)	0.179 (9)	0.147 (8)	0.043 (6)	0.033 (5)	0.070 (7)
Co1	0.0380 (2)	0.0324 (2)	0.0258 (2)	0.00821 (17)	0.00750 (16)	0.01238 (17)
N1	0.0318 (12)	0.0416 (14)	0.0325 (14)	0.0070 (11)	0.0047 (10)	0.0198 (12)
N2	0.0401 (13)	0.0410 (14)	0.0260 (13)	0.0049 (11)	0.0074 (10)	0.0140 (11)
N3	0.0559 (19)	0.062 (2)	0.073 (2)	0.0101 (16)	0.0259 (17)	0.0368 (19)
O1	0.0474 (12)	0.0295 (10)	0.0286 (11)	0.0133 (9)	0.0070 (9)	0.0097 (9)
O2	0.0571 (14)	0.0414 (13)	0.0326 (12)	0.0172 (11)	0.0017 (11)	0.0073 (10)
O3	0.0421 (11)	0.0367 (11)	0.0274 (11)	0.0148 (9)	0.0073 (9)	0.0087 (9)
O4	0.0451 (12)	0.0452 (13)	0.0476 (14)	0.0200 (11)	0.0099 (11)	0.0178 (11)
O5	0.0461 (12)	0.0337 (11)	0.0325 (11)	0.0097 (9)	0.0121 (9)	0.0144 (9)

### Geometric parameters (Å, °)

**Table:**

C1—O1	1.308 (3)	C16—C17	1.340 (6)
C1—C6	1.411 (4)	C16—H16	0.9300
C1—C2	1.420 (4)	C17—C18	1.402 (6)
C2—O2	1.371 (4)	C17—H17	0.9300
C2—C3	1.377 (4)	C18—C19	1.369 (4)
C3—C4	1.403 (5)	C18—H18	0.9300
C3—H3	0.9300	C19—O4	1.360 (4)
C4—C5	1.346 (5)	C19—C20	1.426 (5)
C4—H4	0.9300	C20—O3	1.308 (4)
C5—C6	1.433 (4)	C21—O4	1.438 (4)
C5—H5	0.9300	C21—H21A	0.9600
C6—C7	1.414 (5)	C21—H21B	0.9600
C7—N1	1.307 (4)	C21—H21C	0.9600
C7—H7	0.9300	C22—O2	1.410 (4)
C8—C13	1.396 (5)	C22—H22A	0.9600
C8—C9	1.396 (4)	C22—H22B	0.9600
C8—N1	1.411 (4)	C22—H22C	0.9600
C9—C10	1.373 (6)	C23—N3	1.137 (4)
C9—H9	0.9300	C25—C24	1.4738 (12)
C10—C11	1.378 (7)	C25—H25A	0.9600
C10—H10	0.9300	C25—H25B	0.9600
C11—C12	1.372 (5)	C25—H25C	0.9600
C11—H11	0.9300	C24—N4	1.1532 (11)
C12—C13	1.399 (5)	Co1—O1	1.884 (2)
C12—H12	0.9300	Co1—O3	1.884 (2)
C13—N2	1.420 (4)	Co1—O5	2.030 (2)
C14—N2	1.299 (4)	Co1—N1	1.890 (2)
C14—C15	1.422 (5)	Co1—N2	1.885 (3)
C14—H14	0.9300	Co1—C23	1.858 (3)
C15—C20	1.420 (5)	O5—H51	0.8500
C15—C16	1.423 (5)	O5—H52	0.8500
			
O1—C1—C6	124.8 (3)	O4—C19—C18	125.5 (3)
O1—C1—C2	117.7 (3)	O4—C19—C20	113.5 (3)
C6—C1—C2	117.5 (3)	C18—C19—C20	121.0 (3)
O2—C2—C3	125.0 (3)	O3—C20—C15	124.8 (3)
O2—C2—C1	113.5 (3)	O3—C20—C19	117.5 (3)
C3—C2—C1	121.5 (3)	C15—C20—C19	117.7 (3)
C2—C3—C4	120.3 (3)	O4—C21—H21A	109.5
C2—C3—H3	119.9	O4—C21—H21B	109.5
C4—C3—H3	119.9	H21A—C21—H21B	109.5
C5—C4—C3	120.1 (3)	O4—C21—H21C	109.5
C5—C4—H4	120.0	H21A—C21—H21C	109.5
C3—C4—H4	120.0	H21B—C21—H21C	109.5
C4—C5—C6	121.2 (3)	O2—C22—H22A	109.5
C4—C5—H5	119.4	O2—C22—H22B	109.5
C6—C5—H5	119.4	H22A—C22—H22B	109.5
C1—C6—C7	122.6 (3)	O2—C22—H22C	109.5
C1—C6—C5	119.5 (3)	H22A—C22—H22C	109.5
C7—C6—C5	117.9 (3)	H22B—C22—H22C	109.5
N1—C7—C6	126.0 (3)	N3—C23—Co1	178.5 (3)
N1—C7—H7	117.0	C24—C25—H25A	109.5
C6—C7—H7	117.0	C24—C25—H25B	109.5
C13—C8—C9	119.4 (3)	H25A—C25—H25B	109.5
C13—C8—N1	114.1 (3)	C24—C25—H25C	109.5
C9—C8—N1	126.4 (3)	H25A—C25—H25C	109.5
C10—C9—C8	119.9 (4)	H25B—C25—H25C	109.5
C10—C9—H9	120.0	N4—C24—C25	169.6 (8)
C8—C9—H9	120.0	C23—Co1—O3	92.27 (12)
C9—C10—C11	120.6 (4)	C23—Co1—O1	91.05 (13)
C9—C10—H10	119.7	O3—Co1—O1	84.29 (9)
C11—C10—H10	119.7	C23—Co1—N2	90.44 (13)
C12—C11—C10	120.7 (4)	O3—Co1—N2	95.12 (11)
C12—C11—H11	119.6	O1—Co1—N2	178.41 (10)
C10—C11—H11	119.6	C23—Co1—N1	89.65 (12)
C11—C12—C13	119.5 (4)	O3—Co1—N1	178.03 (10)
C11—C12—H12	120.2	O1—Co1—N1	95.21 (10)
C13—C12—H12	120.2	N2—Co1—N1	85.34 (12)
C8—C13—C12	119.8 (3)	C23—Co1—O5	178.58 (11)
C8—C13—N2	114.3 (3)	O3—Co1—O5	89.01 (9)
C12—C13—N2	125.8 (3)	O1—Co1—O5	89.68 (10)
N2—C14—C15	125.9 (3)	N2—Co1—O5	88.83 (11)
N2—C14—H14	117.1	N1—Co1—O5	89.08 (10)
C15—C14—H14	117.1	C7—N1—C8	121.7 (3)
C20—C15—C14	122.1 (3)	C7—N1—Co1	124.9 (2)
C20—C15—C16	119.0 (3)	C8—N1—Co1	113.1 (2)
C14—C15—C16	118.8 (3)	C14—N2—C13	121.8 (3)
C17—C16—C15	121.4 (4)	C14—N2—Co1	125.6 (2)
C17—C16—H16	119.3	C13—N2—Co1	112.7 (2)
C15—C16—H16	119.3	C1—O1—Co1	125.53 (19)
C16—C17—C18	120.6 (3)	C2—O2—C22	117.8 (3)
C16—C17—H17	119.7	C20—O3—Co1	126.2 (2)
C18—C17—H17	119.7	C19—O4—C21	118.4 (3)
C19—C18—C17	120.3 (3)	Co1—O5—H51	112.8
C19—C18—H18	119.9	Co1—O5—H52	109.2
C17—C18—H18	119.9	H51—O5—H52	107.5

### Hydrogen-bond geometry (Å, °)

**Table:**

*D*—H···*A*	*D*—H	H···*A*	*D*···*A*	*D*—H···*A*
O5—H51···O1^i^	0.85	2.18	2.913 (3)	145.
O5—H51···O2^i^	0.85	2.24	2.959 (3)	142.
O5—H52···O3^i^	0.85	2.28	2.926 (3)	133.
O5—H52···O4^i^	0.85	2.10	2.883 (3)	153.

Symmetry codes: (i) −*x*+1, −*y*+1, −*z*+1.
